# Health professionals’ perceptions about their clinical performance and the influence of audit and feedback on their intentions to improve practice: a theory-based study in Dutch intensive care units

**DOI:** 10.1186/s13012-018-0727-8

**Published:** 2018-02-17

**Authors:** Wouter T. Gude, Marie-José Roos-Blom, Sabine N. van der Veer, Dave A. Dongelmans, Evert de Jonge, Jill J. Francis, Niels Peek, Nicolette F. de Keizer

**Affiliations:** 1 Department of Medical Informatics, Academic Medical Center, Amsterdam Public Health Research Institute, University of Amsterdam, Amsterdam, The Netherlands; 2 National Intensive Care Evaluation (NICE) Foundation, Amsterdam, The Netherlands; 3 Centre for Health Informatics, Division of Informatics, Imaging and Data Sciences, Faculty of Biology, Medicine and Health, Manchester Academic Health Science Centre, The University of Manchester, Manchester, UK; 4 Health eResearch Centre, The Farr Institute of Health Informatics Research, Manchester, UK; 5 Department of Intensive Care Medicine, Academic Medical Center, University of Amsterdam, Amsterdam, The Netherlands; 6 Department of Intensive Care Medicine, Leiden University Medical Center, Leiden, The Netherlands; 7 Centre for Health Services Research, City University of London, London, UK; 8 NIHR Greater Manchester Primary Care Patient Safety Translational Research Centre, Manchester Academic Health Science Centre, The University of Manchester, Manchester, UK

**Keywords:** Intensive care, Medical audit, Feedback, Quality improvement, Quality indicators

## Abstract

**Background:**

Audit and feedback aims to guide health professionals in improving aspects of their practice that need it most. Evidence suggests that feedback fails to increase accuracy of professional perceptions about clinical performance, which likely reduces audit and feedback effectiveness. This study investigates health professionals’ perceptions about their clinical performance and the influence of feedback on their intentions to change practice.

**Methods:**

We conducted an online laboratory experiment guided by Control Theory with 72 intensive care professionals from 21 units. For each of four new pain management indicators, we collected professionals’ perceptions about their clinical performance; peer performance; targets; and improvement intentions before and after receiving first-time feedback. An electronic audit and feedback dashboard provided ICU’s own performance, median and top 10% peer performance, and improvement recommendations. The experiment took place approximately 1 month before units enrolled into a cluster-randomised trial assessing the impact of adding a toolbox with suggested actions and materials to improve intensive care pain management. During the experiment, the toolbox was inaccessible; all participants accessed the same version of the dashboard.

**Results:**

We analysed 288 observations. In 53.8%, intensive care professionals overestimated their clinical performance; but in only 13.5%, they underestimated it. On average, performance was overestimated by 22.9% (on a 0–100% scale). Professionals similarly overestimated peer performance, and set targets 20.3% higher than the top performance benchmarks. In 68.4% of cases, intentions to improve practice were consistent with actual gaps in performance, even before professionals had received feedback; which increased to 79.9% after receiving feedback (odds ratio, 2.41; 95% CI, 1.53 to 3.78). However, in 56.3% of cases, professionals still wanted to improve care aspects at which they were already top performers. Alternatively, in 8.3% of cases, they lacked improvement intentions because they did not consider indicators important; did not trust the data; or deemed benchmarks unrealistic.

**Conclusions:**

Audit and feedback helps health professionals to work on aspects for which improvement is recommended. Given the abundance of professionals’ prior good improvement intentions, the limited effects typically found by audit and feedback studies are likely predominantly caused by barriers to translation of intentions into actual change in clinical practice.

**Trial registration:**

ClinicalTrials.gov NCT02922101. Registered 26 September 2016.

**Electronic supplementary material:**

The online version of this article (10.1186/s13012-018-0727-8) contains supplementary material, which is available to authorized users.

## Background

Audit and feedback (A&F) interventions provide health professionals with a summary of their clinical performance over a specified period of time and are a widely used approach to improve quality of care [1]. A Cochrane review of 140 A&F studies concluded that feedback is effective, but with only a median 4.3% absolute improvement (interquartile range 0.5 to 16%) [1]. The reasons behind this limited and variable effect are only partially understood, and further research into the underlying mechanisms through which A&F brings about change is needed to increase its effects [2, 3].

A&F is thought to work because it improves the accuracy with which health professionals self-assess their performance [4]. If they make a negative assessment of their clinical performance by comparing their performance to a target, professionals will develop intentions to improve practice [5]. Therefore, those informed by A&F may be better able to focus their time and resources available for quality improvement more efficiently on those care aspects that need it most. However, previous studies have shown that feedback messages are rejected when recipients do not trust the data, disagree with benchmarks, consider improvement unfeasible, or do not consider the clinical topic an important aspect of care quality [6–9]. An empirical study showed that health professionals ignored between a third and half of the improvement recommendations when confronted with feedback on multiple quality indicators [6]. This may indicate that health professionals already have certain perceptions about their clinical performance before receiving feedback, and that many times, feedback fails to change those perceptions. In turn, this potentially prevents professionals from developing intentions to improve their practice even if improvement is recommended, or, leads to retaining intentions to improve while there may not be room for improvement [10]. We refer to this problem as the information–intention gap. Intentions are essential for initiating behaviour [11]. A lack of correspondence between health professionals’ intended improvement targets and recommended improvement targets may therefore play an important role in explaining the limited effects of A&F interventions [3, 12].

We designed a theory-based experiment to investigate health professionals’ perceptions about their clinical performance and the influence of feedback on their intentions to change clinical practice.

## Methods

### Theoretical framework

Our theoretical framework which we based on Control Theory [5] is published in the study protocol [13]. It assumes that health professionals continuously self-assess their clinical performance by comparing their performance to a target. If they make a negative assessment (i.e. perceived clinical performance < target), the theory predicts that health professionals will develop intentions to take improvement actions and continue these actions until their performance matches or exceeds the target. However, if they observe a discrepancy that is too great, or lack the skills or knowledge on how to improve, recipients may disregard the discrepancy or lower their target to make it more achievable [14, 15]. A&F may influence professionals’ improvement intentions by changing their underlying perceptions about their own performance and the appropriateness of targets.

### Study setting

**Fig. 1:**
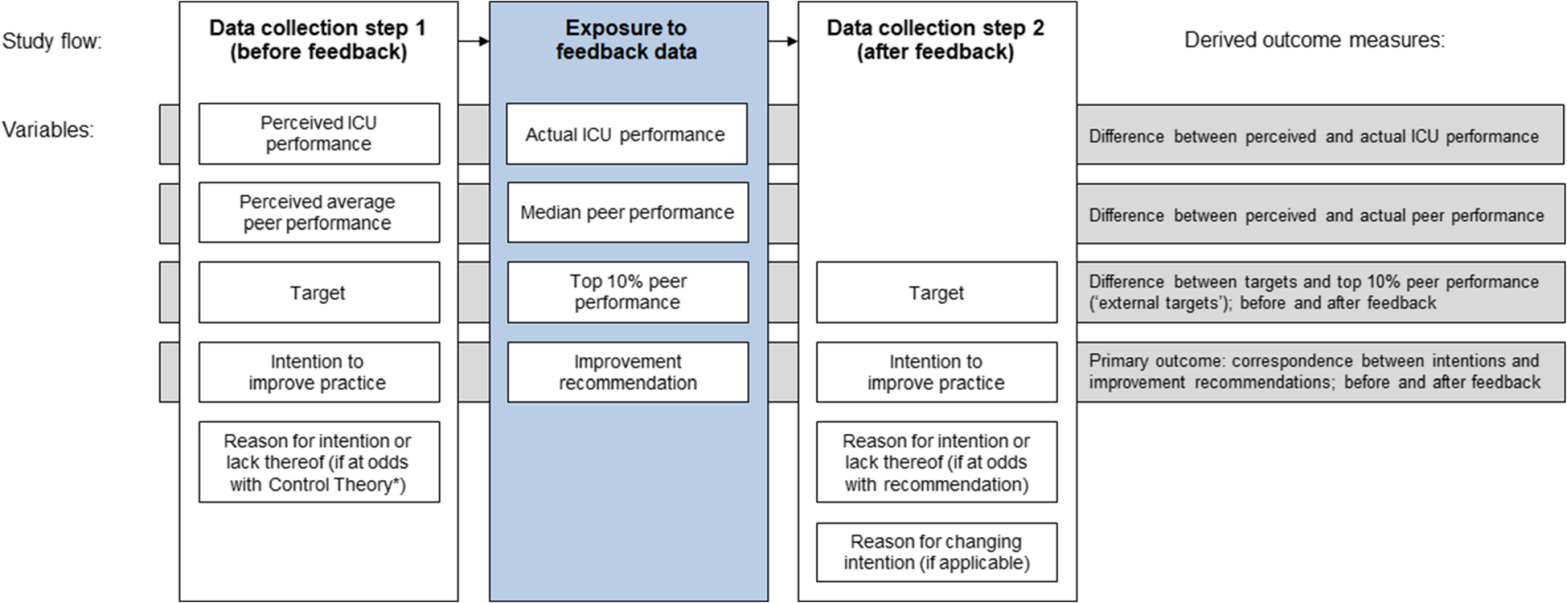
Study flow. Measured and delivered variables for each of four indicators and outcome measures. We collected 288 observations (4 indicators × 72 ICU professionals). *We considered an intention to be at odds with Control Theory if participants had no intention despite a negative self-assessment (i.e. perceived performance < target) or they had improvement intention despite a positive self-assessment (i.e. perceived ICU performance ≥ target)

The study was a laboratory experiment conducted with individual health professionals working in Dutch intensive care units. It was part of a larger study involving a cluster-randomised controlled trial (cRCT) in which teams received periodic feedback on four newly developed pain management indicators through an online interactive dashboard [13]. The cRCT aimed to assess the impact of adding a toolbox with suggested actions and materials to improve intensive care pain management (registered at ClinicalTrials.gov with reference NCT02922101). The laboratory experiment used an adapted version of the dashboard to elicit the individual professionals’ clinical performance perceptions and improvement intentions before and after receiving first-time feedback (Fig. 1). It took place approximately 1 month before enrolling into the cRCT. During the experiment, the toolbox was inaccessible; all participants accessed the same version of the dashboard. Users logging into the dashboard for the first time automatically started the experiment. The study protocol has been previously published [13] and is summarised below.

### Participants

We invited all 83 intensive care professionals who were members of the local quality improvement teams from all 21 ICUs participating in the trial. Personalised invitation emails were sent with up to two reminders. Teams usually consisted of three to five members and included intensivists, nurses, and managers.

### Intervention description

The dashboard provides feedback on four pain management indicators (screenshot available in Additional file 1). The indicator set was recently developed in close collaboration with ICU professionals using an extensive rating and consensus procedure. For each indicator, the dashboard lists the performance score achieved by the ICU (e.g. percentage of patients per shift whose pain is measured), the median score of all participating ICUs, the average score achieved by the top 10% best performing ICUs [16], and a performance assessment represented by a ‘traffic light’ coloured icon; all calculated over the most recent 3 months. Green icons (good performance) are assigned to performance scores above or slightly under the top 10% peer performance. If not green, yellow icons (room for improvement) are assigned to scores above or slightly under the median peer performance; red icons (improvement recommended) are assigned otherwise (for the precise thresholds for assigning icons we refer to the study protocol [13]). From the dashboard overview, users can drill down to see detailed performance information, using trend charts displaying their own and peer past performance over time, performance scores grouped by most relevant patient subgroups (e.g. only surgical patients; only patients during night shifts), and lists of individual patient numbers with an indication whether or not the indicator was violated during a shift. Additional static information about the indicators are available to users, namely, their operationalisation, goal, relation to quality, definitions, inclusion and exclusion criteria, type (process or outcome), and unit of observation.

### Data collection

Data collection took place in two steps (Fig. 1). In the first step, the description of the four indicators and their static information were presented, but measured performance information was withheld. Participants were asked to estimate for each indicator their own ICU’s performance score (perceived clinical performance; range 0–100%) and the average score across Dutch ICUs (perceived peer performance; range 0–100%); fill out the minimum performance score they would consider ‘good’ performance (target; range 0–100%); and whether or not they would perform actions to improve upon the selected indicator (intention to improve practice; range yes/no). If the underlying Control Theory hypothesis was violated (e.g. negative self-assessment but no intention to improve), participants were asked to explain their choice using a predefined list of reasons or in free text (Additional file 2). The provided predefined reasons were developed guided by theoretical behaviour change frameworks [11, 17] and previous work [6, 18].

In the second step, participants were additionally exposed to all detailed performance information for the indicators including their own current performance score; own past performance scores; and the median and top 10% peer performance scores. Like in step 1, participants were asked—but this time based on the information at hand—what their performance target was (range; 0–100%) and if they intended to improve practice (range yes/no). If improvement intentions did not correspond with the improvement recommendation presented in the dashboard (e.g. room for improvement but no intention to improve), participants were again asked to explain their choice using the same list of predefined reasons as in step 1, extended with three reasons relating to feedback rejection (Additional file 2) or free text.

Finally, if there were discrepancies between improvement intentions in the first and second step (e.g. initially participants did not develop intention to improve on a specific indicator, but after receiving feedback they did), participants were asked what feedback elements drove them to change (measured performance score were higher/lower than expected; benchmarks were higher/lower than expected; there was a green/yellow/red icon; other [free text]).

### Outcome measures

The primary outcome was the proportion of improvement intentions set by participants that corresponded with the improvement recommendations. We considered an improvement intention to correspond with the recommendation when a participant reported an intention to improve upon an indicator with room for improvement (i.e. red or yellow icon), or had no intention to improve an indicator without room for improvement (i.e. green icon). Secondary outcomes were the difference between perceived clinical performance (before receiving feedback) and measured performance; difference between performance targets set by participants (before receiving feedback) and the external targets determined by the feedback; change in performance targets after receiving feedback; and reasons for not intending to improve on indicators despite a negative self-assessment (i.e. perceived clinical performance < target) and vice versa.

### Statistical analysis

Descriptive statistics were calculated as appropriate for all variables of interest. We compared performance scores and targets chosen by participants (i.e. perceived clinical performance; perceived peer performance; target) to those that resulted from the audit (i.e. actual clinical performance; median peer performance; external target [top 10% peer performance]), and calculated Pearson’s correlations. We used *t* tests to test whether the differences were significant. To assess the influence of feedback on the correspondence of participants’ improvement intentions with the improvement recommendations we used mixed-effects logistic regression analysis, using a binary ‘feedback received’ covariate. We added a random intercept for ‘participating professional’ to adjust for correlations between repeated observations within participants, added random intercepts for ‘ICU’, and ‘quality indicator’ to adjust for clustering effects within ICUs and around quality indicators. We additionally assessed whether clinical performance perceptions and improvement intentions differed between professional roles (i.e. nurses, intensivists, managers, or others) for each quality indicator (Additional file 3).

## Results

Seventy-two individual professionals (response rate, 87%) from all 21 intensive care units accepted our invitation and participated in the laboratory experiment. The majority included nurses and intensivists (Table 1). There were three (14%) university hospitals, ten (48%) teaching hospitals, and eight (38%) non-teaching hospitals represented in our study. On average, the units held 16 (SD, 9.1) hospital beds and admitted 1240 (SD, 772.5) patients each year. Participants were each confronted with four pain management indicators, yielding a total of 288 observations.

**Table 1:** Characteristics of individual intensive care professionals invited to participate in the study

Characteristic	Value*
Gender	
Male	41 (56.9)
Female	31 (43.1)
Mean age in years (SD)	47.1 (8.9)
Mean clinical experience in years (SD)	24.0 (10.0)
Discipline	
ICU nurse	28 (38.8)
Intensivist	25 (34.7)
Manager	6 (8.3)
Other (e.g. quality officer)	13 (18.1)
Coordinating function	37 (51.4)
Time spent on direct patient care	
< 25%	20 (27.8)
25–50%	7 (9.7)
50–75%	14 (19.4)
> 75%	31 (43.1)

*Values are numbers (percentages) unless indicated otherwise

*SD* standard deviation, *ICU* intensive care unit

### Clinical performance perceptions and improvement intentions before receiving feedback

Table 2 displays participants’ clinical performance perceptions and improvement intentions, and how these varied between indicators. These did not differ between professional roles (Additional file 3). Prior to receiving feedback, participants estimated their performance across all indicators at a median of 70% (IQR, 50 to 85) and average peer performance at a median of 70% (IQR, 60 to 80). These variables correlated strongly (*r* = 0.85, *p* ≤ 0.001), indicating that participants typically estimated themselves as average performers. Participants set their targets at a median 90% (IQR, 80 to 95%). This resulted in 211 (73.3%) negative and 77 (26.7%) positive self-assessments of their performance, with a mean perceived performance-target gap of 18.2% (SD, 20.6).

**Table 2:** Actual performance and recommendations (upper rows) and perceived clinical performance and intentions to improve practice (lower rows)

	All quality indicators (*n* = 288)	Measuring pain (*n* = 72)	Acceptable pain scores (*n* = 72)	Repeating pain measurements (*n* = 72)	Normalised pain scores (*n* = 72)
*Actual performance and recommendations*					
Performance, median (IQR)	41.4 (11.4–82.6)	66.2 (55.7–83.8)	84.9 (82.4–89.6)	12.9 (4.6–22.2)	9.3 (4.1–21.1)
Top 10% peer performance, median (IQR)	74.5 (38–92.4)	91.2 (89.6–95.2)	92.5 (90.8–94)	38 (38–52.5)	31.7 (30.8–42.3)
Improvement recommendation					
Good performance, *n* (%)	71 (24.7%)	15 (20.8%)	40 (55.6%)	8 (11.1%)	8 (11.1%)
Room for improvement, *n* (%)	112 (38.9%)	28 (38.9%)	30 (41.7%)	26 (36.1%)	28 (38.9%)
Improvement recommended, *n* (%)	105 (36.5%)	29 (40.3%)	2 (2.8%)	38 (52.8%)	36 (50%)
*Perceived performance and improvement intentions*					
Perceived performance, median (IQR)	70 (50–85)	86.5 (70–95.8)	75 (60–80)	60 (40–80)	60 (40–80)
Perceived peer performance, median (IQR)	70 (60–80)	80 (70–85)	75 (60–80)	60 (50–80)	62.5 (50–80)
Target [before feedback], median (IQR)	90 (80–95)	90 (88.8–95)	87.5 (80–95)	90 (80–100)	80 (75–90)
Target [after feedback], median (IQR)	90 (75–90)	90 (90–95)	90 (85–90.6)	80 (60–90)	70 (50–90)
Intention to improve [before feedback], *n* (%)	230 (79.9%)	49 (68.1%)	58 (80.6%)	62 (86.1%)	61 (84.7%)
Intention to improve [after feedback], *n* (%)	239 (83%)	58 (80.6%)	43 (59.7%)	69 (95.8%)	69 (95.8%)

In 235 cases (81.6%), the presence or absence of improvement intentions was consistent with our theoretical framework based on the negative or positive self-assessment participants made by comparing perceived performance to their target. Participants had the intention to improve their performance on 230 (79.9%) indicator values; 194 (84.3%) of these followed from a negative self-assessment. In the remaining 36 (15.7%) cases, participants positively self-assessed but still had the intention to improve their performance because they considered the indicator an essential aspect of intensive care quality that should always be targeted for improvement (*n* = 29) and that they deemed it easy to improve on the indicator (*n* = 7). There were 58 (20.1%) indicator values for which participants did not have the intention to improve their performance; 41 (70.7%) of these followed from a positive self-assessment. In the remaining 17 (29.3%) cases, participants negatively self-assessed but deemed improvement unfeasible (*n* = 10), considered the indicator not an important aspect of intensive care (*n* = 6), or lacked time and resources (*n* = 1).

### Correspondence of clinical performance perceptions and improvement intentions with actual performance and improvement recommendations

**Fig. 2:**
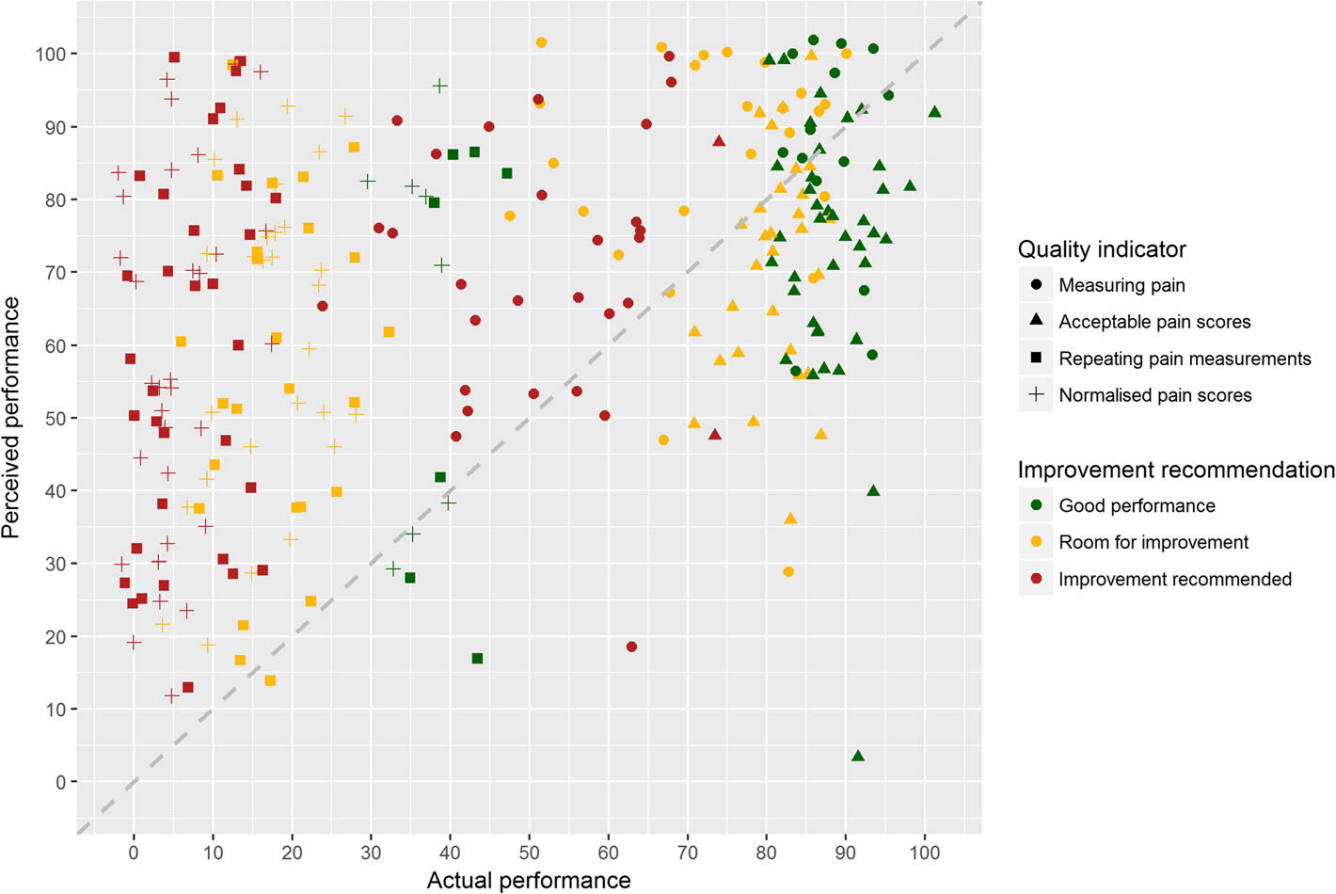
Scatter plot of intensive care professionals’ perceived clinical performance compared to their actual performance (above diagonal line = overestimation; below = underestimation)

Median actual performance was 41.4% (IQR, 11.4 to 82.6), but varied between indicators from 9.3 to 84.9 (Table 2). Hence, participants overestimated their own performance on average by 22.9% (95% CI, 18.1 to 27.7). When an accurate estimation was defined as within 15% range, 94 (32.6%) estimations were accurate, 155 (53.8%) were overestimated, and 39 (13.5%) were underestimated (Fig. 2). Participants’ estimations of their own performance correlated moderately with actual performance (*r* = 0.38, *p*≤ 0.001). Peer performance was also overestimated by 23.5% (95% CI, 18.1 to 27.7) and had a weak correlation (*r* = 0.28, *p*≤ 0.001) with actual median peer performance. The mean performance of the top 10% best performers, used in this study as the external target and to define ‘good performance’, was 74.5% (IQR, 38.0 to 92.4). This means that targets set by participants were on average 20.3% (95% CI, 16.7 to 23.8) higher than the external targets; participants’ own targets and the feedback’s external targets correlated weakly (*r* = 0.14, *p* = 0.029). Although—on average—participants’ perceived performance and set targets were equally higher than the measured performance and external targets; this was often inconsistent within the same observation (e.g. participants could have overestimated performance on an indicator by 50% while setting their target at the same level as the external target or vice versa). As a result, only 186 times (64.6%) the positive or negative self-assessments participants made by comparing perceived performance to their target corresponded with the external assessment.

Based on comparisons between actual performance and the external targets, there were 71 (24.7%) cases of ‘good performance’; in 112 (38.9%) cases, there was room for improvement, and in 105 (36.5%) cases, improvement was recommended. Prior to receiving feedback about this, 197 (68.4%) of participants’ intentions already corresponded with the feedback’s recommendations. Specifically, participants had the intention to improve upon 178 (82.0%) ‘room for improvement’ and ‘improvement recommended’ indicator values, and did not have the intention to improve upon 19 (26.7%) ‘good performance’ indicators (Fig. 2 and Table 3).

**Table 3:** Correspondence between intensive care professionals’ intentions and improvement recommendations before and after receiving feedback

	Intentions corresponding with improvement recommendations		Absolute risk difference	Odds ratio* (95% CI)	*p* value
	Before feedback	After feedback			
All quality indicators (*n* = 288)	197 (68.4%)	230 (79.9%)	11.5%	2.41 (1.53 to 3.78)	< 0.001
Improvement recommendation					
Good performance (*n* = 71)	19 (26.7%)	31 (43.7%)	17.0%	3.88 (1.39 to 10.87)	0.010
Room for improvement and improvement recommended (*n* = 217)	178 (82.0%)	199 (91.7%)	9.7%	4.36 (1.94 to 9.79)	< 0.001

*Adjusted for clustering with random effects for each individual professional, ICU and quality indicator

In the 217 cases in which there was room for improvement, participants who had the intention to improve overestimated their own performance similarly to those who did not (30.1 versus 36.7% overestimation; *p* = 0.247), but did set higher targets relative to the external targets (32.3 versus 21.1% higher than the external targets; *p* = 0.012). In the other 71 cases of ‘good performance’, both participants who did and those who did not have the intention to improve similarly overestimated performance (4.6% underestimation versus 5.2% overestimation; *p* = 0.248) and set similar targets relative to the external targets (16.2 versus 14.3% higher than the external targets; *p* = 0.823).

### Change in improvement intentions and performance targets after receiving feedback

**Fig. 3:**
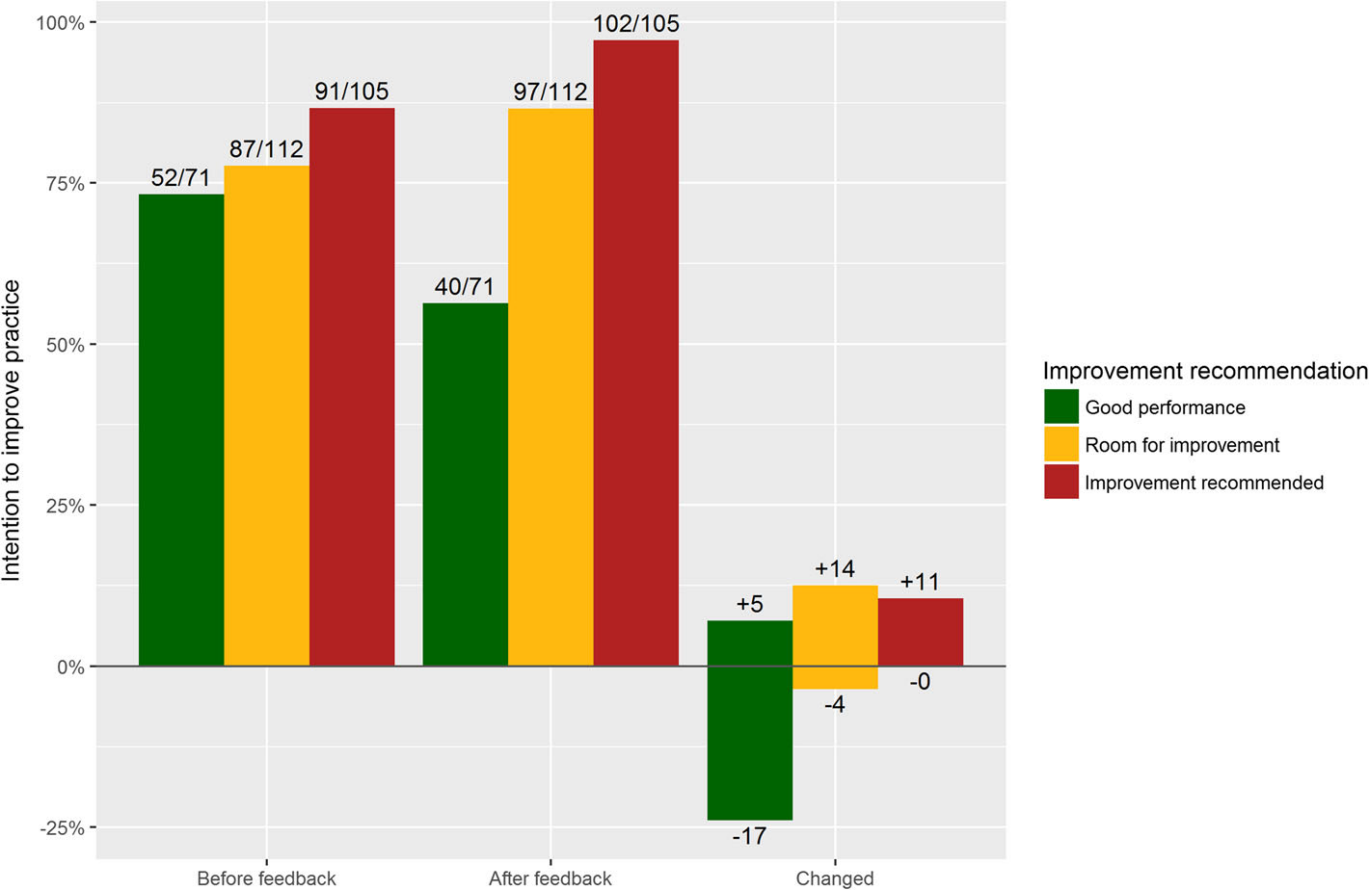
Bar chart of intensive care professionals’ intentions to improve practice before and after receiving clinical performance feedback

After receiving feedback on their performance and seeing external performance targets, participants changed their original intention in 51 (17.7%) of the cases. In 41 (15.5%) cases, this happened when their original intention was at odds with the feedback’s improvement recommendation. Namely, for 17 indicator values which the feedback reported ‘good performance’ participants withdrew their original improvement intention; for 25 ‘room for improvement’ or ‘improvement recommended’ indicator values, participants developed new intentions. In addition, however, participants also changed 9 (3.4%) of their intentions that were already in line with the recommendation: they withdrew four intentions for ‘room for improvement’ indicators and developed review new intentions for ‘good performance’ indicators. Overall, the correspondence between participants’ intentions and the improvement recommendations increased to 208 (79.9%) cases (Fig. 3 and Table 3). Regression analysis showed that participants’ intentions became more than twice as likely to follow the improvement recommendations (OR, 2.41; 95% CI, 1.53 to 3.78; *p* < 0.001) (Table 3). The main reason reported for developing new intentions was that the own performance score was lower than expected (*n* = 29). Other reasons were that they were following the feedback’s recommendation (*n* = 3) and that the peer performance was higher than expected (*n* = 2). The main reason for withdrawing intentions was that the own performance score was higher than expected (*n* = 16). Other reasons were that the peer performance was lower than expected (*n* = 3) or that they were following the feedback’s recommendation (*n* = 3).

In total, participants ignored 58 (20.1%) of the improvement recommendations provided by the feedback. For 40 (56.3%) ‘good performance’ indicator values, they still had the intention to improve because they thought their performance score was too low (*n* = 20), considered the indicator an essential aspect of intensive care quality that should always be targeted for improvement (*n* = 14), or deemed it easy to improve on the indicator (*n* = 4). For 18 (8.3%) ‘room for improvement’ or ‘improvement recommended’ indicator values, participants had no intention to improve because they considered their measured performance score inaccurate (*n* = 7); the indicator not an important aspect of intensive care (*n* = 6); benchmarks unrealistic or unachievable (*n* = 3); or improvement unfeasible (*n* = 2).

Participants did downward adjust their targets to a median 90% (IQR, 75 to 90). These new targets were on average 13.9% (95% CI, 10.1 to 17.67) higher than the external targets; their correlation was stronger (*r* = 0.53, p≤ 0.001) than before receiving feedback. There were no significant differences in targets and intentions between nurses, intensivists, and managers (Additional file 3).

## Discussion

Our study showed that 53.8% of the time, intensive care professionals substantially overestimated their clinical performance; only 13.5% of the time they underestimated it. Professionals also overestimated peer performance and set their targets much higher than the external targets used in this study. In 81.6% of cases, professionals’ improvement intentions could be predicted by Control Theory [5] based on the performance self-assessments professionals had made by comparing their perceived performance score to their target. Already 68.4% of those intentions corresponded with the improvement recommendations even before they had received feedback on their clinical performance; which further increased to 79.9% after receiving feedback. The feedback made it more than twice as likely that professionals’ intentions followed the improvement recommendations. Our findings therefore seem to confirm that feedback increases the accuracy with which health professionals self-assess their performance; which is the principal hypothesised mechanism through which A&F is thought to work [1, 4].

The intensive care professionals attributed changes in their intentions particularly to the performance score they received, not to the median or top 10% peer performance benchmarks. This suggests that professionals did adopt new insights about their own performance, but less so about appropriate performance targets. Although professionals in this study did slightly downward adjust their targets based on the feedback they received, they still set them higher than the top 10% peer performance. As a result participants had the intention to improve half of the cases in which they were already top performers. Although improvement could still be achievable, setting reasonable targets should help professionals to prioritise their improvement activities. Study periods in A&F trials are often limited, and health professionals typically have to choose between multiple care aspects to focus on [19, 20]. Many hypotheses exist about how performance comparators may trigger more reactions to the feedback, such as using achievable benchmarks instead of medians [21]. At the moment, there is conflicting evidence about this hypothesis [16, 22], and our results demonstrate that intensive care professionals often aim for something higher regardless of which comparator is delivered.

In 8.3% of cases, intensive care professionals had no intention to improve upon quality indicators for which the feedback recommended improvement. In comparison, in a similar experiment in cardiac rehabilitation, professionals ignored one third of the cases for which the feedback recommended improvement [6]. The difference might relate to the cognitive load for health professionals which was smaller in the current study in terms of the number and variety of targeted behaviours [21, 23], and other differences in design and content of the feedback. In our study, professionals rejected feedback because they considered some quality indicators as not an important aspect of intensive care, did not trust the measured own performance score, or considered the benchmarks unrealistic.

### Strengths and limitations

The principal strength of our study is the extensive use of Control Theory [5] as a basis for our study design. Although there is growing recognition that theory should play a central role in the design and evaluation of A&F interventions [2], explicit use of theory remains scarce [24, 25]. We tested the hypothesis that A&F increases the correspondence between health professionals’ intentions and improvement recommendations; this hypothesis is typically assumed to be true in A&F studies but has not, to the best of our knowledge, been evaluated empirically. Which threshold is used to determine such recommendations is a design choice to be made by A&F designers; in this study, we used top 10% peer performance. Using a different target would lead to a different correspondence between intentions and recommendations. However, it would unlikely affect the relationship we found between providing the feedback and professionals changing their intentions.

The generalisability of our findings with respect to health professionals’ improvement intentions may be limited due to the clinical topic and setting, namely pain management in ICUs. ICU professionals traditionally work in a data-rich environment and are early adopters of A&F systems. This could have resulted in our participants being more experienced and set higher targets than health professionals from other domains. At the same time the extent to which indicators are under ICU professionals’ control may differ between professionals. For example, performing pain measurements is typically a nursing task, while intervening to achieve acceptable pain scores falls under the responsibility of intensivists. Therefore, participants might have estimated their ICU’s performance more accurately and set more informed targets if we would have asked them to respond as a team. The fact that we found no significant differences in performance estimations or improvement intentions between professional roles for any of the indicators might reflect the strong team mentality and shared responsibilities in ICU patient care. As this might be different in other settings, it is however a pertinent point to consider whether feedback actually reaches the health professionals whose behaviour is targeted for change and whether the recommendations arising from the feedback make it clear who is responsible for taking action [26].

We developed the dashboard carefully considering the latest evidence, theory, and design suggestions and involved intensive care professionals in the process [13]. This likely contributed to the fact that in 91.7% of cases, feedback convinced participants to change practice. The indicator set was developed in close collaboration with experts and pilot tested to ensure that the indicators are consistent with professionals’ goals and priorities and under their control [13, 23]. It seems however inevitable that some professionals have concerns about the importance of certain indicators. Lack of trust in data quality, often identified as a barrier to change [15, 27], might in this study have particularly related to the perception that the targeted behaviour is performed in practice (e.g. patients’ pain is measured each shift) but not recorded electronically. We undertook various data quality assurance efforts [28] and provided patient-level data and subgroup analyses to increase transparency [29]. More intensive measures might be required to ensure professionals recognise the importance of indicators and trust in the data, e.g. through verbal feedback and when feedback is discussed by teams rather than individuals [1, 6]. In addition we delivered multiple performance comparators to prevent benchmarks being perceived as unrealistically high; namely median, top 10% peer performance, and own past performance. Delivering multiple comparators is at odds with recent suggestions for A&F design because it could create ambiguity in what should be achieved [23]. Our findings however show that multiple comparators worked well despite possible ambiguities. To reduce ambiguity, we delivered traffic light colour-coded benchmark comparisons.

We conducted our study with intensive care professionals in a laboratory setting shortly before enrolment in a cRCT in which they received feedback on the four new pain management indicators for the first time. By aligning the experiment with the cRCT, we were able to deliver professionals’ real clinical performance data and obtain a high response rate of nearly 90%, while using the same dashboard and data collection methods. Laboratory experiments in the field of A&F are scarce but enable a great opportunity to gain detailed insights into professionals’ decision making that allows us to advance implementation science while reducing research waste [30].

### Unanswered questions and future research

Given the great presence of health professionals’ intentions to improve the care they provide, the limited effectiveness often found in A&F studies is likely the result of barriers to translating intentions into actual change in clinical practice. Future research should focus on overcoming those barriers, and less so on convincing professionals to improve practice. The cRCT following the current study will (1) reveal whether teams target less indicators for improvement in practice, e.g. due to prioritisation or resource limitations, and (2) determine the effectiveness of augmenting the dashboard with an action implementation toolbox to address the gap between intentions and actions [13].

## Conclusion

Health professionals often overestimate their clinical performance and rarely underestimate it. In addition, they are typically optimistic about the performance of peers and achievable targets. Nevertheless, their prior intentions to improve practice often already correspond to actual gaps in clinical performance. Feedback further increases this correspondence because it allows professionals to self-assess their performance more accurately. However, professionals still have a tendency to want to improve upon care aspects at which they are already top performers. In a tenth of cases, professionals lack improvement intentions because they do not consider some indicators an essential aspect of care quality, do not trust the data, or deem the benchmarks unrealistic. Given the abundance of health professionals’ good improvement intentions, it is likely that the limited effects typically found by audit and feedback studies are predominantly caused by barriers to translation of intentions into actual change in clinical practice. Interventions should focus on overcoming those barriers, and less so on convincing professionals to improve practice.

## Additional files

Additional file 1: Screenshot dashboard (translated from Dutch). (PDF 137 kb)

Additional file 2: Predefined reasons to be asked if hypotheses posed by Control Theory are violated. (PDF 37 kb)

Additional file 3: Linear (for perceived clinical performance and targets) and logistic (for intention to improve) regression analysis results assessing differences between different professional roles for each quality indicator. The reason to explore this is that response might be affected by the level of control participants have over specific quality indicators. For example, nurses might have more control over measuring pain than intensivists and therefore estimate performance or set targets more realistically than others, or develop different intentions to improve. The analyses show that this is however not the case; which might be explained by the shared responsibility for patient care and close collaboration in quality teams in intensive care units in comparison to general wards. (PDF 68 kb)

## Abbreviations

A&F: Audit and feedback
cRCT: Cluster-randomised controlled trial
ICU: Intensive care unit
IQR: Interquartile range
NICE: National Intensive Care Evaluation foundation
SD: Standard deviation
