# Supplementary material for: Health professionals’ perceptions about their clinical performance and the influence of audit and feedback on their intentions to improve practice: a theory-based study in Dutch intensive care units
Source: Implement Sci. 2018 Feb 17;13:33. doi: 10.1186/s13012-018-0727-8 (PMC5816547; doi:10.1186/s13012-018-0727-8)
Supplement: Supplementary file 1 — Screenshot dashboard (translated from Dutch). (PDF 137 kb) [file 13012_2018_727_MOESM1_ESM.pdf]

Additional file 1. Screenshot dashboard (translated from Dutch).

Dashboard actionable indicators

NICE foundation website | NICE Online | Data dictionary | Data uploads | Help | username(logout)

Pain | Blood | Antibiotics | Ventilation

Action plan overview →

Quality indicator	Own ICU	Median	Top 10%	Target	Actions
Performing pain measurements each shift	**89,0%**	67,0%	90,1%	100,0%	0
Acceptable pain scores	**76,2%**	85,0%	90,9%	80,0%	0
Repeating pain measurements with unacceptable score within 1 hour	**44,9%**	13,0%	55,2%	100,0%	2
Unacceptable pain scores normalised within 1 hour	**36,6%**	9,2%	29,0%	60,0%	0

Details | Patients | Info | Action plan

Average performance in most recent 3 months:

Own ICU	Median	Top 10%	Previous period
89,0% (good performance)	67,0%	90,1%	-0,1%

Performance over time:

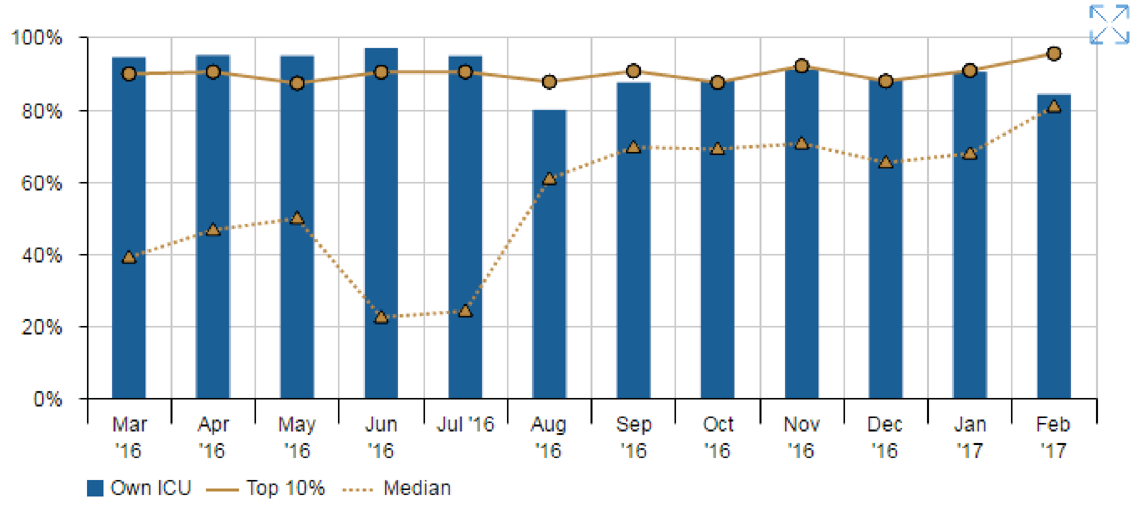

Operationalisation:
Patient shifts during which pain was measured at least once.

Period:
1 December 2016 t/m 28 February 2017

Population in this period:

Own ICU:	180 admissions
All ICUs:	3840 admissions in 12 ICUs

Colour thresholds based on all ICUs:

- 81,1% - 100,0% Good performance
- 60,3% - 81,1% Room for improvement
- 0,0% - 60,3% Improvement recommended

Actions:
0 actions in progress
0 actions implemented
