# Supplementary material for: Health professionals’ perceptions about their clinical performance and the influence of audit and feedback on their intentions to improve practice: a theory-based study in Dutch intensive care units
Source: Implement Sci. 2018 Feb 17;13:33. doi: 10.1186/s13012-018-0727-8 (PMC5816547; doi:10.1186/s13012-018-0727-8)
Supplement: Supplementary file 2 — Predefined reasons to be asked if hypotheses posed by Control Theory are violated. (PDF 37 kb) [file 13012_2018_727_MOESM2_ESM.pdf]

Additional file 2. Predefined reasons to be asked if hypotheses posed by Control Theory are violated.

**Hypothesis violation**	**Predefined reason**
Negative self-assessment but no improvement intention	This indicator is not an important/relevant aspect of intensive care
	Actions will not improve our performance score on this indicator
	We lack the resources/time/knowledge to take action for this indicator
	Me or my colleagues cannot be motivated to take action for this indicator
	The benchmark (median/top 10%) is unrealistic/unfeasible (step 2 only)
	The measured performance score for our ICU is inaccurate (step 2 only)
Positive self-assessment and improvement intention	This indicator is an essential aspect of quality of intensive care
	It is easy to improve our performance score on this indicator
	Our performance is too low (step 2 only)
