# Supplementary material for: Health professionals’ perceptions about their clinical performance and the influence of audit and feedback on their intentions to improve practice: a theory-based study in Dutch intensive care units
Source: Implement Sci. 2018 Feb 17;13:33. doi: 10.1186/s13012-018-0727-8 (PMC5816547; doi:10.1186/s13012-018-0727-8)
Supplement: Supplementary file 3 — Linear (for perceived clinical performance and targets) and logistic (for intention to improve) regression analysis results assessing differences between different professional roles for each quality indicator. The reason to explore this is that response might be affected by the level of control participants have over specific quality indicators. For example, nurses might have more control over measuring pain than intensivists and therefore estimate performance or set targets more realistically than others, or develop different intentions to improve. The analyses show that this is however not the case; which might be explained by the shared responsibility for patient care and close collaboration in quality teams in intensive care units in comparison to general wards. (PDF 68 kb) [file 13012_2018_727_MOESM3_ESM.pdf]

Additional file 3. Linear (for perceived clinical performance and targets) and logistic (for intention to improve) regression analysis results assessing differences between different professional roles for each quality indicator. The reason to explore this is that response might be affected by the level of control participants have over specific quality indicators. For example, nurses might have more control over measuring pain than intensivists and therefore estimate performance or set targets more realistically than others, or develop different intentions to improve. The analyses show that this is however not the case; which might be explained by the shared responsibility for patient care and close collaboration in quality teams in intensive care units in comparison to general wards.

	**Measuring pain (n=72)**		**Acceptable pain scores (n=72)**		**Repeating pain measurements (n=72)**		**Normalised pain scores (n=72)**	
Perceived clinical performance [1]	β (95% CI)	p	β (95% CI)	p	β (95% CI)	p	β (95% CI)	p
Intensivists vs nurses	-6.25 (-15.99 to 3.50)	0.205	-3.95 (-12.82 to 4.93)	0.378	0.07 (-13.99 to 14.12)	0.993	-0.21 (-12.82 to 12.41)	0.974
Managers vs nurses	-2.14 (-18.11 to 13.83)	0.790	4.26 (-10.25 to 18.77)	0.560	0.51 (-22.57 to 23.59)	0.965	7.52 (-13.20 to 28.25)	0.471
Other vs nurses	-8.27 (-3.70 to 20.24)	0.172	6.01 (-4.81 to 16.83)	0.272	13.20 (-3.97 to 30.36)	0.130	15.29 (-0.11 to 30.69)	0.052
Target [before feedback] [2]	β (95% CI)	p	β (95% CI)	p	β (95% CI)	p	β (95% CI)	p
Intensivists vs nurses	-2.13 (-5.67 to 1.41)	0.234	2.93 (-2.97 to 8.82)	0.325	-0.66 (-7.11 to 5.78)	0.838	-1.79 (-9.43 to 5.85)	0.641
Managers vs nurses	0.16 (-5.56 to 5.87)	0.956	-0.35 (-9.96 to 9.27)	0.943	-0.99 (-11.53 to 9.55)	0.852	0.30 (-12.26 to 12.86)	0.962
Other vs nurses	-0.40 (-4.70 to 3.89)	0.852	-1.80 (-9.01 to 5.42)	0.621	-2.50 (-10.51 to 5.50)	0.535	4.06 (-5.54 to 13.67)	0.401
Target [after feedback] [3]	β (95% CI)	p	β (95% CI)	p	β (95% CI)	p	β (95% CI)	p
Intensivists vs nurses	0.86 (-3.48 to 5.20)	0.694	0.47 (-2.87 to 3.82)	0.778	8.17 (-3.24 to 19.57)	0.158	9.52 (-2.77 to 21.80)	0.127
Managers vs nurses	1.67 (-5.52 to 8.87)	0.644	1.47 (-4.11 to 7.05)	0.600	4.45 (-14.50 to 23.40)	0.641	4.76 (-15.62 to 25.15)	0.642
Other vs nurses	4.13 (-1.24 to 9.49)	0.129	-0.20 (-4.27 to 3.87)	0.923	10.62 (-3.25 to 24.50)	0.131	9.65 (-5.26 to 24.56)	0.201
Intention to improve [before feedback] [4]	OR (95% CI)	p	OR (95% CI)	p	OR (95% CI)	p	OR (95% CI)	p
Intensivists vs nurses	1.65 (0.35 to 7.67)	0.525	2.99 (0.49 to 18.14)	0.233	1.02 (0.09 to 11.61)	0.986	2.87 (0.37 to 22.06)	0.310
Managers vs nurses	2.74 (0.21 to 35.50)	0.440	0.62 (0.07 to 5.33)	0.665	0.15 (0.00 to 4.41)	0.269	0.39 (0.04 to 3.90)	0.422
Other vs nurses	1.12 (0.22 to 5.64)	0.892	1.38 (0.23 to 8.18)	0.724	1.03 (0.07 to 16.24)	0.981	0.68 (0.09 to 5.27)	0.715
Intention to improve [after feedback] [5]	OR (95% CI)	p	OR (95% CI)	p	OR (95% CI)	p	OR (95% CI)	p
Intensivists vs nurses	0.13 (0.01 to 1.27)	0.080	2.29 (0.03 to 14.16)	0.836	>95% of participants had intentions to improve upon these indicators; not enough variation to perform regression analysis			
Managers vs nurses	1.84 (0.08 to 40.08)	0.697	1.03 (0.03 to 33.96)	0.989				
Other vs nurses	**0.04 (0.00 to 0.62)**	**0.021**	1.89 (0.08 to 43.91)	0.693				

β = coefficient. p = p-value. OR = odds ratio. CI = confidence interval. [1] Adjusted for actual performance. [2] Adjusted for perceived performance. [3] Adjusted for actual performance and median and top 10% benchmarks. [4] Adjusted for target before feedback and perceived performance. [5] Adjusted for target after feedback, actual performance, and median and top 10% benchmarks.
